# Fluorescent Method for the Detection of Biothiols Using an Ag^+^-Mediated Conformational Switch

**DOI:** 10.3390/s19040934

**Published:** 2019-02-22

**Authors:** Han Zhao, Mingjian Chen, Changbei Ma

**Affiliations:** School of Life Sciences, Central South University, Changsha 410083, China; 172511001@csu.edu.cn (H.Z.); chenmingjian@csu.edu.cn (M.C.)

**Keywords:** fluorescence, glutathione, cysteine, 2-aminopurine

## Abstract

In this work, a novel, simple, and time-saving fluorescence approach for the detection of biothiols (glutathione and cysteine) was developed by employing a DNA probe labeled with 2-aminopurine. As an adenine analogue, 2-aminopurine exhibits high fluorescence intensity that can be rapidly quenched in the presence of DNA. In the presence of Ag^+^, the fluorescence increased significantly, which was a result of the formation of cytosine–Ag^+^–cytosine base pairs and the release of 2-aminopurine. Upon addition of either glutathione or cysteine, the structure of cytosine–Ag^+^–cytosine was disrupted, a product of the stronger affinity between biothiols and Ag^+^. As a result, the 2-aminopurine-labeled DNA probe returned to its former structure, and the fluorescence signal was quenched accordingly. The detection limit for glutathione and cysteine was 3 nM and 5 nM, respectively. Furthermore, the determination of biothiols in human blood serum provided a potential application for the probe as a diagnostic tool in clinical practice.

## 1. Introduction

In recent years, the detection of biothiols, i.e., thiol-containing amino acids and peptides, as well as glutathione (GSH) and cysteine (Cys) has attracted significant attention due to the important effects these compounds exhibit in physiological systems [1,2]. As the richest endogenous cellular thiol, GSH plays a crucial role in several biological processes, including intracellular reduction environments, defense mechanisms against toxins, neutralization of free radicals and peroxides, as well as signal transport and gene regulation [3,4,5,6,7,8]. Abnormal levels of GSH have been reported to be partly responsible for various physiological conditions, such as Alzheimer’s disease, diabetes, HIV, premature aging, liver damage, and various cancer types [9,10,11]. Cys represents a critical, extracellular reducing agent via the formation of disulfide bonds. It further serves as the rate-limiting precursor for GSH and taurine synthesis [12]. Recent studies have shown that cysteine deficiency is closely related to Parkinson’s disease, reduced growth, liver damage, psoriasis, skin lesions, lethargy, leukocyte loss, etc. [13,14]. Hence, the development of a facile, rapid, and yet sensitive method for the detection of biothiols in complicated biological samples is of great importance for monitoring disease states in clinical diagnosis.

A variety of techniques have been proposed for the determination of biothiols, including high performance liquid chromatography (HPLC), colorimetric methods, electrochemiluminescence, surface enhanced Raman scattering (SERS), and mass spectrometry (MS) [15,16,17,18]. Despite promising results displayed by these methods, deficiencies remain, including the need for sophisticated sample preprocessing procedures, expensive equipment, professional instrument operation, long reaction times, as well as tedious sample modifications. Such requirements tremendously limit the overall range of applications using conventional techniques. Recently, attention has increasingly focused on fluorescence-based approaches in an effort to develop a time-saving process that also features high sensitivity [19,20,21,22], ease of operation, and lower detection limits. For this purpose, Xu et al. developed a molecular beacon-based fluorescence assay for the detection of glutathione and cysteine [23]. Meanwhile, Wang et al. established a label-free fluorescent probe for the detection of biothiols based on graphene oxide and a ruthenium complex [24]. Furthermore, Wang et al. employed a nanomaterials-based fluorescent sensor for the determination of biothiols [2,25,26]. However, all of these methods require a complex probe design, often dependent on extensive synthetic work to produce the corresponding probe material.

2-Aminopurine (2AP) represents a fluorophore that is similar to adenine in structure but exhibits high fluorescence even without the presence of another detectable species. Once incorporated into DNA, the fluorescence of 2-aminopurine dramatically decreases; this results from its stacking with ambient nucleotide bases which induce electron transfer and have no need for any exterior quencher [27,28,29,30]. Cytosine may form a complex with Ag^+^ to form a steady cytosine-Ag^+^-cytosine complex with high affinity [31]. Therefore, this paper proposes a novel strategy for the quantification of biothiols based on the competitive binding of Ag^+^ to biothiols and cytosine–cytosine (C–C) mismatched base pairs utilizing a 2-aminopurine-labeled DNA probe. Furthermore, the method we present herein was successfully applied to the detection of biothiols in human serum samples, providing a promising application for clinical diagnosis.

## 2. Materials and Methods

### 2.1. Materials and Reagents

The sequence of the DNA probe was as follows: 5′-CCCCC2APCCCCC-3′ (C10-2AP). The sequence was purchased from Sangon Biological Engineering Technology & Services (Shanghai, China). Glutathione (GSH), Cysteine (Cys), and other amino acids were purchased from Source leaf Biotechnology Co., Ltd. (Shanghai, China). Silver nitrate (AgNO_3_) and 3′-(N-morpholino) propanesulfonic acid (MOPS) were purchased from Sinopharm Chemical Reagent Co., Ltd. (Shanghai, China). Ultrapure water (18.2 MΩ·cm) used in the experiments was obtained from a Milli-Q water purification system (Millipore Corp., Bedford, MA, USA).

### 2.2. Apparatus

The detection of fluorescence intensities was carried on an F-2700 fluorescence spectrophotometer (Hitachi, Japan). The excitation wavelength was set at 310 nm and the fluorescence emission spectra were recorded from 350 nm to 450 nm at room temperature. The slit widths of excitation and emission were both set to 5 nm.

### 2.3. Optimization of the Experimental Conditions

For the sake of achieving the best experimental results, some critical reaction factors were optimized, including the concentration of the DNA probe and Ag^+^. The concentration range of the DNA probe was 50–300 nM. The Ag^+^ concentration range was 0.5 to 2 μM.

### 2.4. Fluorescence Detection of Glutathione and Cysteine

Defined concentrations of GSH and Cys in aqueous solution were prepared freshly before use. At room temperature, different concentrations of biothiols were mixed with 10 mM MOPS (pH = 7.0), 100 nM DNA probe, and 1.2 μM Ag^+^ to obtain a final volume of 100 μL. The resulting mixture was then allowed to react for 10 min. Afterwards, the fluorescence intensity was recorded at a wavelength range of 350 to 450 nm.

### 2.5. Selectivity Assay

A variety of amino acids containing Arg, Tyr, His, Trp, Gly, Ser, Ala, Val, Leu, Ile, Met, Pro, Phe, Thr, Gln, Asn, Asp, and Lys (1 μM) were added to the reaction buffer containing MOPS (10 mM, pH = 7.0), the DNA probe (100 nM), and Ag^+^ (1.2 μM). After reaction for 10 min, the fluorescence intensity was recorded for each amino acid at room temperature.

### 2.6. Sample Assay

For the determination of biothiols, Ag^+^ and the DNA probe were added to a 100-fold diluted human serum sample containing MOPS buffer. The fluorescence was measured thereafter. To test the corresponding recoveries, a given amount of GSH was mixed with the diluted serum for the detection of biothiols using a standard addition method. Fluorescent measurements were carried out as described above. Human serum samples were collected from Xiangya Hospital, Central South University. The study was approved by the Ethics Committee of Central South University, Changsha, China. All people had given informed consent. We declare that the experiments performed in this study comply with the current laws of the People’s Republic of China.

## 3. Results and Discussion

### 3.1. Principle of the Detection of Biothiols

**Scheme 1:**
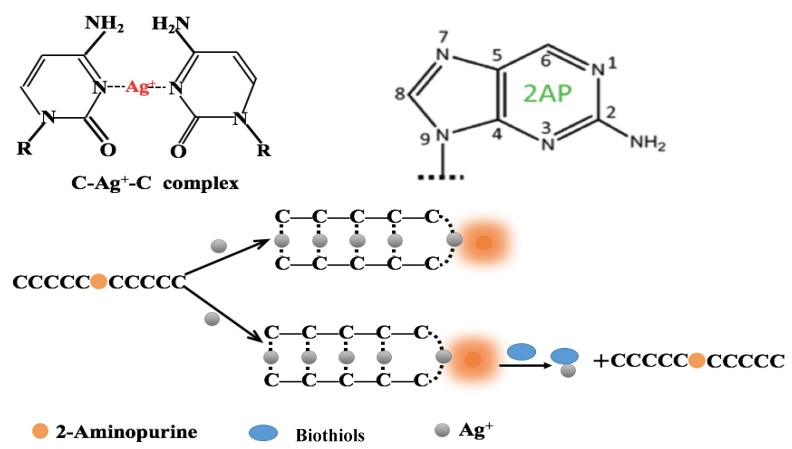
Proposed fluorescence strategy for the determination of biothiols mediated by Ag^+^.

This proposed fluorescence strategy for the determination of biothiols mediated by Ag^+^ can be found illustrated in Scheme 1. The oligonucleotide DNA probe consisted of ten cytosine bases, which embedded 2-aminopurine in the center. The fluorescence intensity of 2-aminopurine decreased significantly when incorporated into a C-rich DNA probe. In the presence of Ag^+^, 2-aminopurine was released by the Ag^+^-mediated formation of C–C mismatch base pairs, resulting in high fluorescence intensity. As a result of the stronger affinity between Ag^+^ and biothiols, the C–Ag^+^–C structure collapsed following the addition of biothiols. This contributed to the oligonucleotide DNA probe returning to its original form; it also resulted in a weak fluorescence signal. Shown to be both time-saving and cost-effective, this fluorescence intensity could be used for the sensitive quantification of biothiols.

### 3.2. Method Feasibility

**Figure 1:**
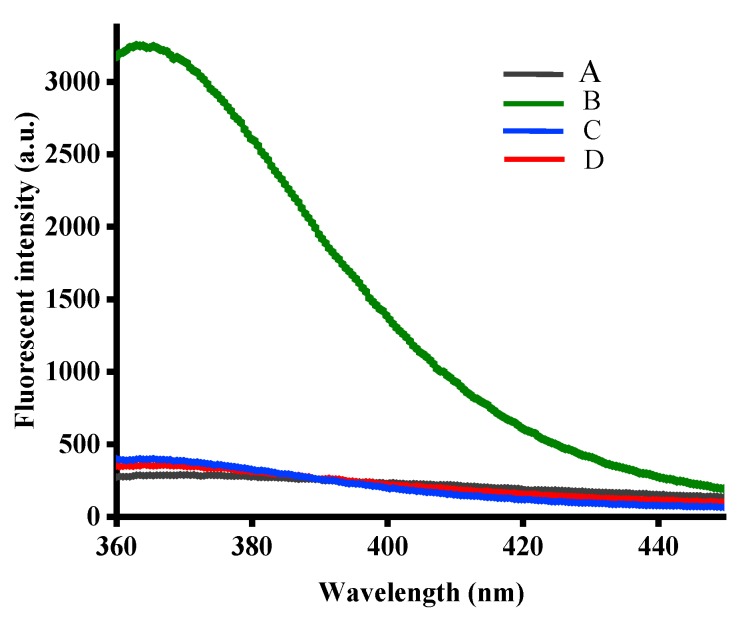
Fluorescence emission spectra of reaction products in the presence of 2AP DNA probe (curve A), Ag^+^ + 2AP DNA probe (curve B), Ag^+^ + 2AP DNA probe + GSH (curve C), Ag^+^ + 2AP DNA probe + Cys (curve D).

To verify the feasibility of the strategy, various experiments under different conditions were carried out. As shown in Figure 1, the C-rich DNA probe exhibited a weak fluorescence signal (curve A) and also featured a fluorescence quenching ability due to base stacking when 2-aminopurine was inserted in the center. After incubation with Ag^+^, a recovery of the fluorescence signal was observed (curve B), which was attributed to the formation of a C–Ag^+^–C structure that led to the release of 2-aminopurine. However, the fluorescence signal decreased sharply when GSH (curve C) or Cys (curve D) was introduced to the reaction system. This was attributable to the stronger binding interactions between biothiols and Ag^+^ when compared with those of the C–Ag^+^–C complex. This resulted in the 2-aminopurine-labeled DNA probe to return to its original structure. Therefore, the results indicated that the 2-aminopurine-labeled DNA probe mediated by Ag^+^ could be successfully used for the analysis of biothiols.

### 3.3. Optimization of Experimental Conditions

**Figure 2:**
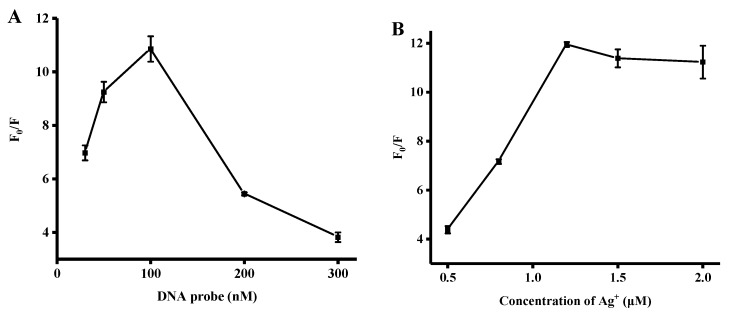
(**A**) Concentration optimization of the 2AP DNA probe. (**B**) Optimization of Ag^+^ concentration. Error bars were estimated from three replicate measurements.

We investigated some experimental conditions that may affect the fluorescent signal in the determination of biothiols, including the concentration of Ag^+^ and DNA probe. Firstly, we optimized the concentration of the DNA probe. As illustrated in Figure 2A, the rate of fluorescence enhancement was demonstrated to increase significantly upon an increase in the concentration of the DNA probe from 30 to 100 nM. However, an excessive amount of the DNA probe led to Ag^+^ binding and the release of 2-aminopurine, which resulted in a decreased F_0_/F value. Here, F_0_ and F represent the corresponding fluorescence intensities in the absence and presence of GSH, respectively. Therefore, an optimal DNA probe concentration of 100 nM was selected.

Moreover, we optimized the Ag^+^ concentration. As shown in Figure 2B, the ratio of fluorescence intensity at 370 nm increased significantly upon further addition of Ag^+^, reaching a plateau at 1.2 μM. Upon an additional amount of Ag^+^, a more stable C–Ag^+^–C complex could be formed and the fluorescence intensity of 2-aminopurine reached a maximum. Thus, a Ag^+^ concentration of 1.2 μM was used throughout the experiments.

### 3.4. Quantification of Biothiols Activity

**Figure 3:**
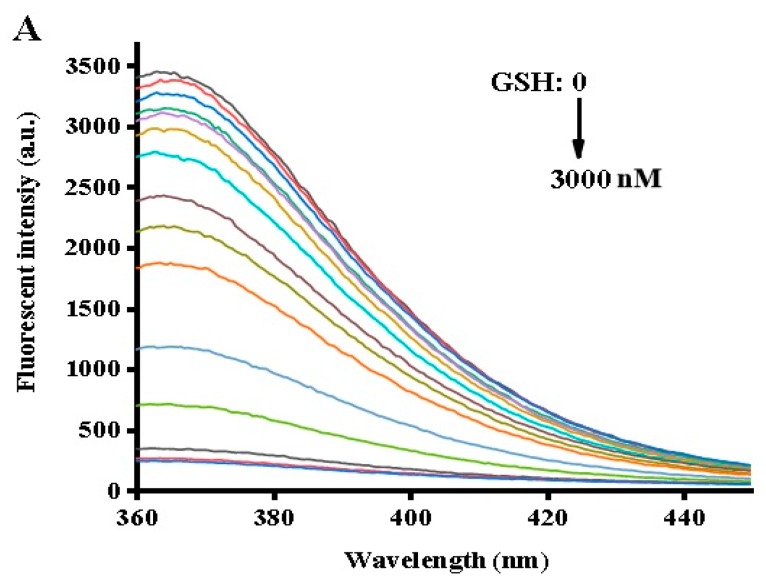

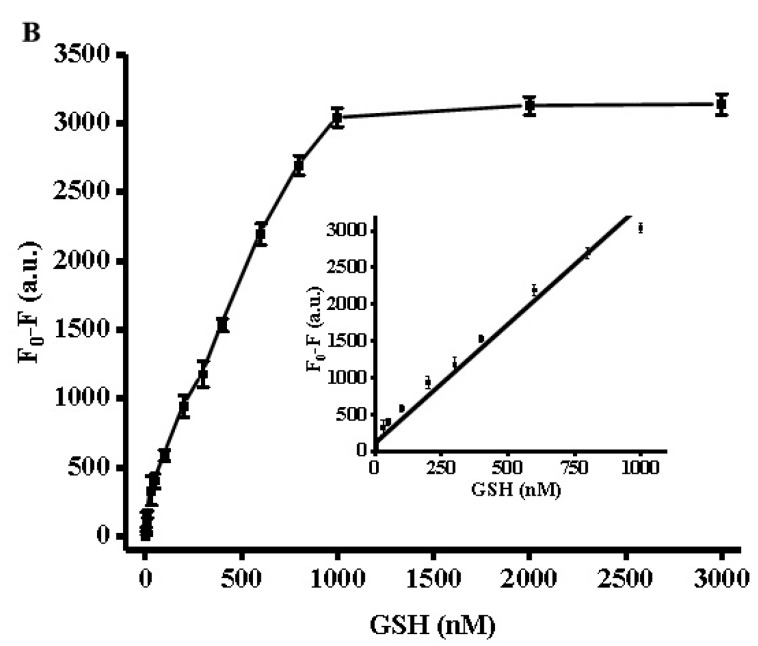
(**A**) Fluorescence spectra with different concentrations of GSH. (**B**) Relationship between fluorescence intensity and GSH concentrations. The inset shows the linearity of the fluorescence intensity with respect to the GSH concentrations. Error bars were estimated from three replicate measurements.

**Figure 4:**
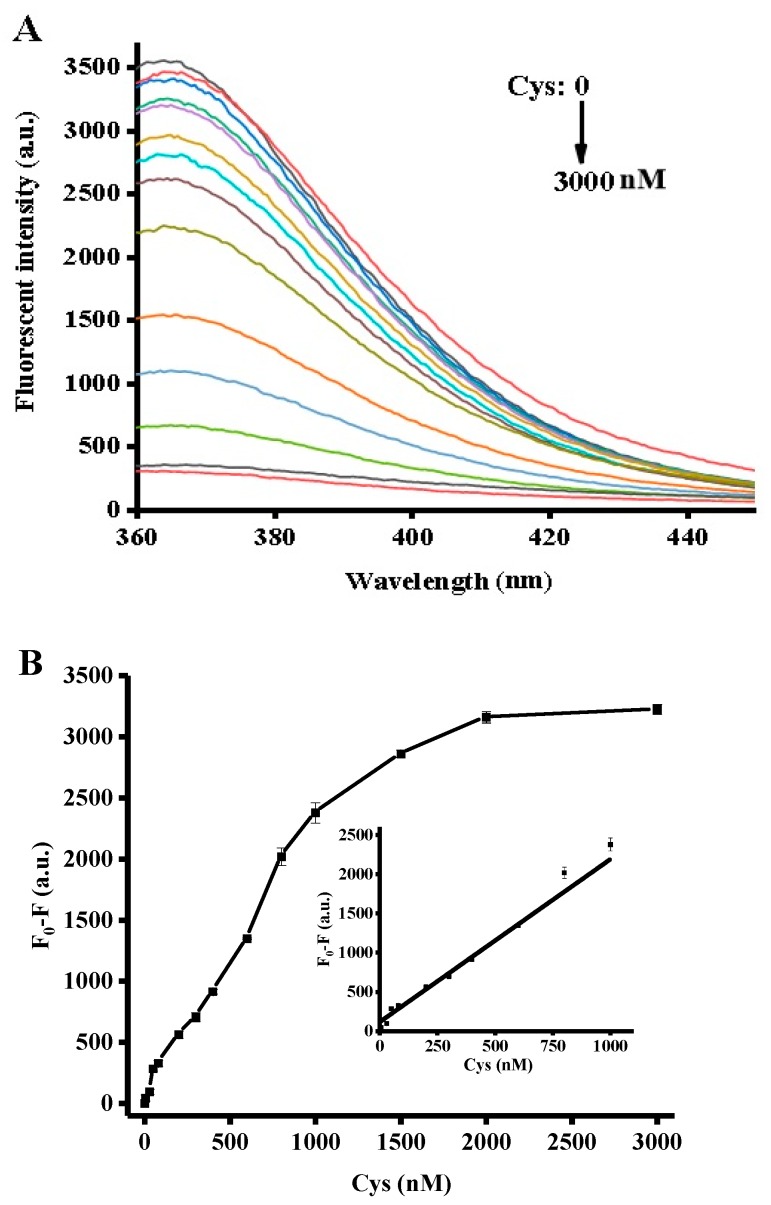
(**A**) Fluorescence spectra with different Cys concentrations. (**B**) Relationship between fluorescence intensity and Cys concentration. The inset shows the linearity of the fluorescence intensity with respect to the Cys concentrations. Error bars were estimated from three replicate measurements.

Using the optimized conditions described above, GSH and Cys measurements were carried out by adding different biothiol concentrations to the reaction system. As seen in the fluorescence spectrum in Figure 3A, different GSH concentrations corresponded to different fluorescence intensities. The fluorescence intensity at 370 nm gradually decreased upon an increase of the GSH concentration from 0 to 3000 nM, reaching a plateau at 2000 nM. As depicted in the inset of Figure 3B, a chart of F_0_–F versus GSH concentrations revealed a good liner correlation at a concentration range of 3 to 1000 nM. Here, F_0_ and F represented the fluorescence intensity in the absence and presence of GSH. The calibration equation could be expressed as F = 2.95727C + 405.71406 (R2 = 0.99071) where F was the peak intensity and C was the concentration of GSH (nM). Cys chelates Ag^+^ ions due to the high affinity of the thiol group to silver ions. Therefore, Cys measurement was carried out similarly to the GSH analysis assay. As shown in Figure 4A,B, the Cys assay showed the same concentration dependence with a linear relationship at a concentration range of 5 to 1000 nM, and the linear regression equation was F = 2.14221C + 136.83356 (R2 = 0.9925), with a detection limit of 5 nM. In comparison with other reported methods for the detection of biothiols (cf. Table 1), the strategy proposed herein proved to be advantageous over conventional methods and was demonstrated to be superior in terms of time-saving operation and sensitivity.

### 3.5. Selectivity for Cysteine and Glutathione

**Figure 5:**
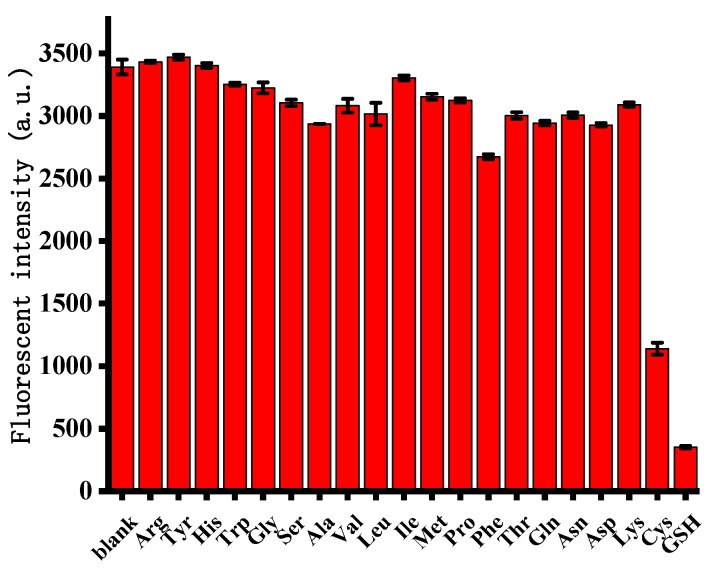
Assay selectivity for GSH and Cys compared with other amino acids. Error bars were estimated from three replicate measurements.

To estimate the selectivity of this strategy, the fluorescence response of this system to a variety of amino acids was evaluated. The concentration of other amino acids was more than 10 times that of GSH and Cys. As shown in Figure 5, they exhibited high fluorescence intensities; however, the fluorescence signal of GSH/Cys was negligible. We speculated that the selectively for GSH and Cys detection could be attributed to the strong affinity of the thiol groups present in GSH and Cys to bind to Ag^+^. Other amino acids without thiol groups were unable to remove Ag^+^ from the C–Ag^+^–C structure. These results suggested that the proposed method featured an excellent selectivity for the determination of biothiols and was further shown to be suitable for applications involving biological samples.

### 3.6. Detection of Biothiols in Serum Samples

To evaluate the applicability of this approach in biological samples, the detection of biothiols in human serum samples was performed. Human serum samples were diluted to ensure that the biothiol concentration was within the detection range of this assay. The corresponding results can be found listed in Table 2. The recoveries of spiked GSH in serum samples were from 97.5% to 102%, demonstrating that this proposed method has the capability to detect biothiols in biological samples.

## 4. Conclusions

In summary, a novel and quencher-free fluorescence approach with high sensitivity and selectivity for the determination of biothiols was developed by employing a 2-aminopurine DNA probe. This strategy relied on the strong affinity between Ag^+^ and biothiols, resulting in the collapse of the C–Ag^+^–C structure and leading to a weak fluorescence intensity. The sensing system featured a linear interval within a concentration range of 3 to 1000 nM for GSH and 5 to 1000 nM for Cys, respectively. Correspondingly, the detection limit was determined to be as low as 3 nM and 5 nM for GSH and Cys, respectively. Several advantages have been identified for the fluorescent DNA probe used throughout our studies, including low cost, short reaction time, and facile operation without need for additional quencher species. Furthermore, the strategy could be successfully applied to the detection of biothiols in human serum samples, further suggesting potential applicability in clinical diagnosis.

## Figures and Tables

**Table 1:** Comparison of different methods for biothiols determination.

Method	Material	Analyte	Linear Range (nM)	LOD (nM)	Reference
ECL	MnO_2_ Nanosheet	GSH	10–2000	3.7	[16]
Colorimetry	Carbon nanodot	GSH	0–7000	300	[32]
Colorimetry	DNAzyme	GSH	40–1500	40	[33]
		Cys	50–650	50	
Fluorescence	G-quadruplex	GSH	50–3600	16	[34]
Fluorescence	Thioflavin T	GSH	30–2000	13.9	[35]
		Cys	20–2500	8.4	
Fluorescence	Graphene oxide and Ru-complex	GSH	0–5000	4.6	[24]
		Cys	0–5000	6.2	
Fluorescence	2-Aminopurine	GSH	3–1000	3	This work
		Cys	5–1000	5	

**Table 2:** Recovery experiments of determination of biothiols in human serum samples.

Sample	Added (μM)	Found (μM)	Recovery (%)
1	0	0.29 ± 0.0034	-
2	0.4	0.68 ± 0.0006	97.5
3	0.5	0.80 ± 0.0031	102
4	0.6	0.88 ± 0.0017	98.3
